# Whole Genome and Single‐Cell RNA Sequencing Reveals Clonal Evolution and Heterogeneity of Secondary Plasma Cell Leukemia: A Case Report

**DOI:** 10.1002/jha2.70215

**Published:** 2026-01-13

**Authors:** Tomotaka Suzuki, Rui Yokomori, Takaomi Sanda, Masaki Ri, Shinsuke Iida

**Affiliations:** ^1^ Department of Hematology and Oncology Nagoya City University Graduate School of Medical Sciences Nagoya Japan; ^2^ Cancer Science Institute of Singapore National University of Singapore Singapore

**Keywords:** clonal evolution, genomic instability, plasma cell leukemia, single‐cell RNA sequencing, whole genome sequencing

## Abstract

Secondary plasma cell leukemia (sPCL) is a rare, aggressive manifestation of multiple myeloma (MM). We report a 75‐year‐old Japanese man with anemia as the chief complaint and IgG‐λ MM that rapidly progressed to sPCL. Whole genome sequencing using Canopy revealed a major clone with a monoallelic *TP53* mutation. During progression, a subclone with a biallelic *TP53* mutation expanded, and the 1q21 copy number increased. Single‐cell RNA‐sequencing identified an emergent PCL population with high CKS1B expression. These data demonstrate genomic instability and clonal evolution during sPCL development, underscoring the need for approaches accounting for tumor heterogeneity in MM.

**Trial Registration**: The authors have confirmed clinical trial registration is not needed for this submission.

## 1 Introduction

Secondary plasma cell leukemia (sPCL) is associated with a poor prognosis; however, the underlying biology and mechanism of progression from multiple myeloma (MM) to sPCL remain largely unknown. We aimed to elucidate the molecular pathogenesis and genetic background of sPCL using whole genome sequencing (WGS) and single‐cell RNA sequencing (scRNA‐seq) analyses of serial samples from a patient with MM who rapidly progressed to sPCL.

## 2 Materials and Methods

### 2.1 Case Presentation

A 75‐year‐old Japanese man presented with anemia as the chief complaint and was diagnosed with IgG‐λ type MM. The patient's clinical information is shown in Supporting Information S1 and Figure S1. Both the International Staging System (ISS) and the revised ISS were classified as Stage II. Bortezomib, melphalan, and prednisolone (VMP) therapy yielded a very good partial response; however, the patient developed sPCL (white blood cell 5400/µL; plasma cells 25%) during the VMP therapy. Treatment with multiple salvage therapies, including anti‐CD38 antibody and immunomodulatory drugs, did not induce a durable response. The patient developed massive pleural effusion (PE) with plasma cell involvement and died 13 months after the diagnosis.

### 2.2 Genetic Abnormalities and Gene Expression Profile

WGS was performed on five samples: (i) peripheral blood (PB) mononuclear cells (PBMCs) at initial diagnosis (“control PBMC”), (ii) bone marrow (BM) sample at initial MM diagnosis (BM_MM), (iii) PB sample at PCL development (PB_PCL), (iv) BM sample at PCL development (BM_PCL), and (v) PE (Figure S1). scRNA‐seq was performed using the 10× Genomics platform for PB_PCL and BM_PCL samples. Further details are provided in Supporting Information S2.

## 3 Results

### 3.1 Single Nucleotide Variants (SNVs) and Clonal Heterogeneity

**FIGURE 1:**
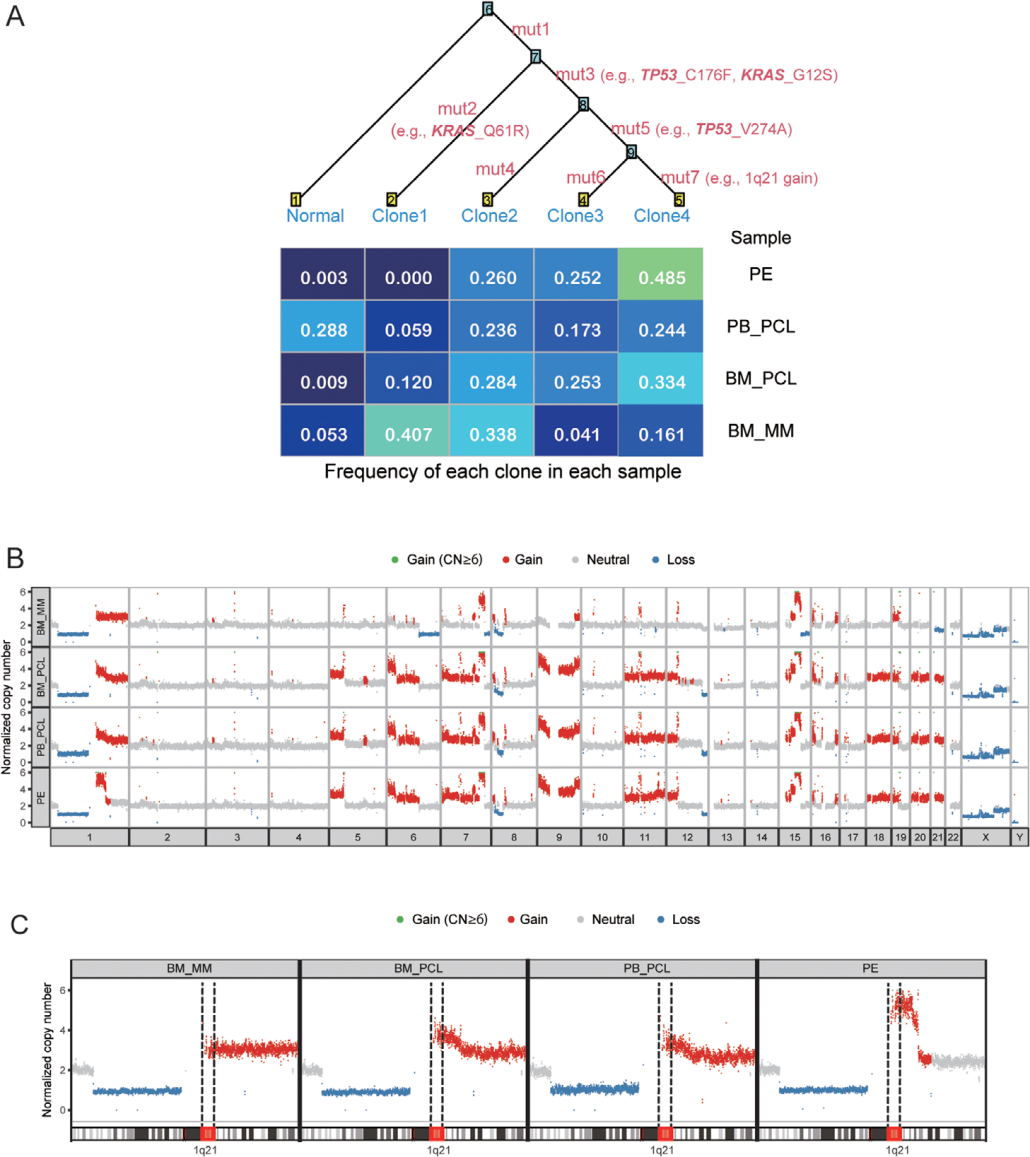
Tumor clonal architecture and phylogenetic tree inferred via Canopy analysis and copy number variations in each sample. (A) Canopy analysis identified four clones (Clones 1–4) based on the selected mutation features (Mutations 1–7). Representative mutations and the frequency of each clone in each sample are shown. (B) Copy number gains and losses for each sample are shown. Several differences were identified between samples. Partial region loss in chromosomes 6q, 7q, 15q, and 21q in BM_MM was not observed after sPCL development. (C) The copy number of Chromosome 1 in each sample is shown. The estimated copy number of the 1q21 region in the BM_MM sample was three, which increased to four in the BM_PCL and PB_PCL samples and six in the PE sample. BM_MM, bone marrow sample obtained at the initial diagnosis of multiple myeloma; BM_PCL, bone marrow sample obtained during the development of plasma cell leukemia; CN, copy number; PB_PCL, peripheral blood sample obtained during the development of PCL; PE, pleural effusion.

SNV analysis revealed that overall profiles were largely identical among all tumor samples (Figures S2A and S3). However, the allele fraction of SNVs, including TP53 mutation, differed among the four samples (Figure S3), which suggests the selection of specific clones in each sample. In fact, clonality analysis with Canopy [1] based on the SNV profile inferred that the dominant clone at the BM_MM stage (Clone 1) was hardly detectable upon progression to sPCL, while a minor clone (Clones 2 and 3) expanded at the time of sPCL development (Figure 1A; Tables S1 and S2). Notably, Clone 2 harbored a single TP53 mutation (C176F), whereas Clone 3 possessed biallelic TP53 mutations (C176F and V274A on separate alleles; Figure S4). Clone 1 harbored KRAS Q61R, whereas Clones 2 and 3 carried KRAS G12S but lacked KRAS Q61R. Clone 3 was also characterized by gain/amplification of the 1q21 region. These clones were also detectable in the BM_MM sample, suggesting the existence of clonal heterogeneity at the initial stage, as well as clonal selection during progression to PCL.

**FIGURE 2:**
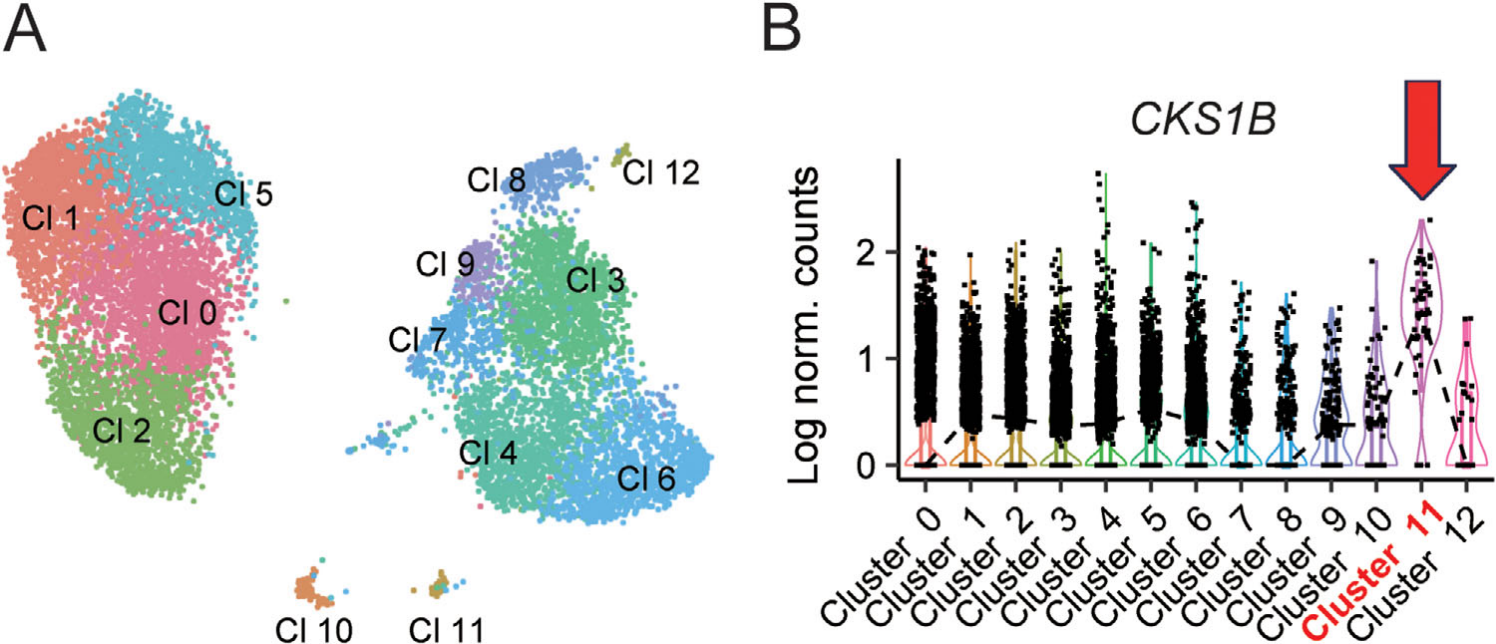
scRNA‐seq analysis of tumor cells in PB and BM samples obtained at the development of sPCL. (A) Uniform manifold approximation and projection plots showing the two distinct clusters of tumor cells in PB and BM samples. Tumors in PB (Clusters 3, 4, 6, 7, 8, 9, 10, 11, and 12) were composed of more clusters than those in BM (Clusters 0, 1, 2, and 5). (B) Violin plots visualizing the expression of *CKS1B*, the representative gene located on 1q21, among the clusters. Cluster 11 showed remarkably high expression of *CKS1B* (indicated by red arrow). BM, bone marrow; FC, fold change; N.S., not significant; PB, peripheral blood; sPCL, secondary plasma cell leukemia; scRNA, single‐cell RNA sequencing.

### 3.2 Structural Variant (SV) and Copy Number Variation (CNV) Analyses

SV evaluation using WGS samples indicated that no notable chromosomal translocations involving the IgH locus with CCND1, MMSET, or MAF were detected. SV number increased with disease progression (Figure 1B and Figure S2B). Notably, the estimated copy number of the 1q region increased from three in the BM_MM sample to a higher level (four in BM_PCL and PB_PCL samples and six in PE_PCL sample) during disease progression (Figure 1C).

### 3.3 scRNA‐seq Analysis

The PB_PCL sample contained non‐myeloma cells, which may limit the accurate evaluation of allele frequency. Hence, we performed a scRNA‐seq on the PB_PCL and BM_PCL samples to distinguish tumor from non‐tumor populations. Integrated analysis of the two samples, followed by cell clustering and visualization using Uniform Manifold Approximation and Projection revealed that tumor cells from each sample formed distinct, non‐overlapping clusters (see Supporting Information S2). The PB_PCL sample had more isolated clusters than the BM_PCL sample, indicating greater heterogeneity in terms of gene expression patterns in the PB_PCL sample (Figure 2A). Notably, Cluster 11, a small cluster in the PB_PCL sample, showed significantly higher CKS1B expression than the other PB_PCL clusters (Figure 2B). Because CKS1B is located in the 1q21 region, this result suggested that a minor clone in the PB sample might have acquired an additional 1q21 copy number gain, which was observed in the PE sample by WGS analysis.

## 4 Discussion

Our integrated analysis revealed several key insights—particularly related to intratumor heterogeneity—that could not have been captured through conventional bulk sequencing alone. First, the total number of detected genetic variants was similar across all samples. However, the Canopy analysis inferred that a minor clone with biallelic *TP53* mutations already existed at the initial MM diagnosis and expanded during progression to sPCL. WGS‐based copy number profiling uncovered extensive chromosomal alterations acquired during sPCL development, and the Canopy analysis suggests that these large‐scale events originated in the *TP53*‐mutated minor clone present at the MM stage. Since CN alterations, in addition to biallelic mutations of *TP53*, can act as driver alterations [2], these chromosomal changes might reflect genomic instability and contribute to the establishment of sPCL.

Next, although there were no notable differences in the overall SNV profile between tumor cells from PB_PCL and BM_PCL, scRNA‐seq revealed that they represent transcriptionally distinct tumor cell populations. This could be owing to a technical batch effect but also suggests that factors other than genetic alterations—such as epigenetic modifications in the microenvironment (i.e., BM vs. PB)—may contribute to the characteristics of PB tumor cells.

Lastly, scRNA‐seq detected a small cluster with high *CKS1B* expression on 1q21 in the PB_PCL sample, suggesting that these cells might acquire additional 1q21 gain. This was consistent with our CNV analysis by WGS (Figure 1B,C). Thus, the small PB_PCL tumor clone is thought to eventually develop into the dominant clone in the PE sample, characterized by higher 1q21 copy numbers in this patient.

Our analysis highlights high genomic instability, significant intratumoral heterogeneity, and stepwise clonal evolution in sPCL biology. These findings underscore the importance of analytical approaches that account for tumor heterogeneity when studying MM. Further studies are required to develop effective treatment strategies for this condition.

## Author Contributions

T.S., R.Y., and T.S. designed the study and wrote the manuscript. M.R. extracted genomic DNA. R.Y. performed the bioinformatics analyses. All the authors contributed to the manuscript and approved the submitted version.

## Funding

This study was supported by the MEXT/JSPS, KAKENHI (18K19960 and 24K02484), and the Japanese Society of Hematology Research Grant 2021.

## Ethics Statement

This study was approved by the Institutional Review Board of Nagoya City University Hospital.

## Consent

The patient provided consent for his samples to be used in the analyses and the results to be published.

## Conflicts of Interest

The authors declare no conflicts of interest.

## Supporting information




**Figure S1**: Clinical course of the patient.

**Table S1**: Genetic Alteration Groups and Corresponding Specific Alterations by Canopy analysis.

**Table S2**: Copy Number Alteration IDs and Corresponding Chromosomal Positions by Canopy analysis.

## Data Availability

Because this study is based on a single patient case, the datasets include potentially identifiable information and cannot be shared to protect patient confidentiality.
