# Supplementary material for: Whole Genome and Single‐Cell RNA Sequencing Reveals Clonal Evolution and Heterogeneity of Secondary Plasma Cell Leukemia: A Case Report
Source: EJHaem. 2026 Jan 13;7(1):e70215. doi: 10.1002/jha2.70215 (PMC12800386; doi:10.1002/jha2.70215)
Supplement: Supplementary file 1 — Figure S1: Clinical course of the patient. [file JHA2-7-e70215-s001.docx]

**Supplementary Material**

## **Doc S1. Supplementary clinical information**

- The patient's past medical history included hypertension and prostate cancer that was resected six years ago without further chemotherapy.
- There was no significant family history of hematologic disorders.
- The laboratory study results of this patient at the initial myeloma diagnosis are shown as below.


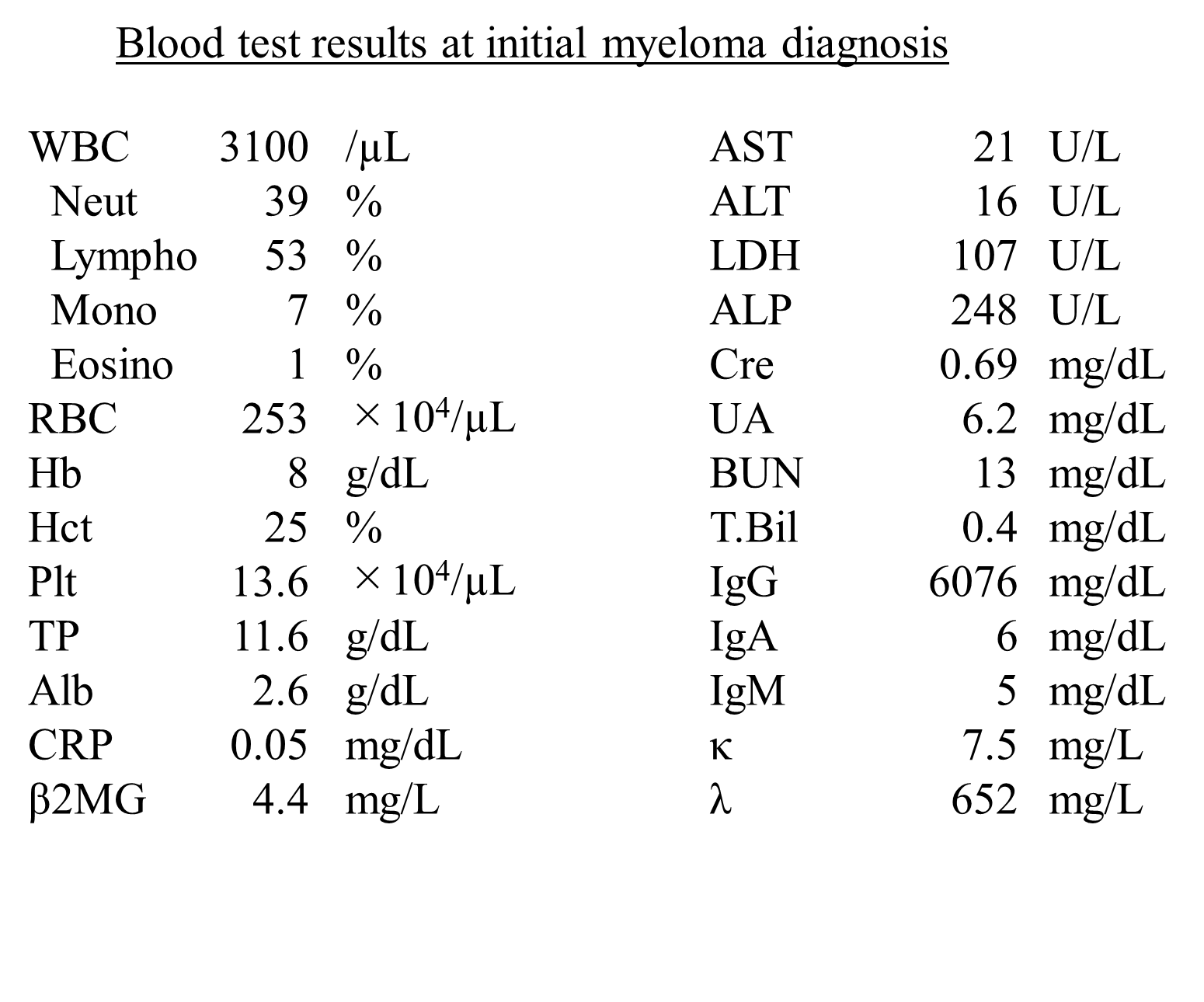


**Doc S2. Supplementary Methods**

### **Extraction of genomic DNA**

Samples obtained from this patient were as follows: i) peripheral blood mononuclear cells (PBMCs) as a control and ii) bone marrow (BM) cells for diagnosis of multiple myeloma (BM_MM), iii) PBMCs and iv) BM cells for diagnosis of PCL (PB_PCL and BM_PCL), and v) pleural effusion (PE) cells. Myeloma cells from BM and pleural effusion were purified by CD138 magnetic sorting, while peripheral blood samples were not purified. Genomic DNA was extracted from these samples using the QIAamp DNA Mini Kit (QIAGEN, Hilden, Germany) according to the manufacturer's instructions.

**Whole-genome sequencing (WGS)**

Library preparation and sequencing were performed by BGI Genomics Co., Ltd. The library was prepared according to the standard WGS library preparation protocol of BGI Genomics Co., Ltd. Sequencing of 150 bp paired-end reads was performed using the DNBSeq platform.

WGS reads were mapped to the reference genome of the hg38 analysis set using speedseq align version 0.1.2 ^1^. Somatic variants (single nucleotide variants and short indels) were detected using GATK Mutect2 version 4.2.6.1 in multi-sample tumor-only mode, with the following options: germline-resource, panel-of-normal, and --f1r2-tar-gz ^2^. GATK FilterMutectCalls was applied to the variants, with the following options: contamination-table and ob-priors. The variants were annotated using SnpEff version 5.1d ^3^. Germline variants were detected for a matched normal sample (the PBMC sample at initial myeloma diagnosis) using GATK HaplotypeCaller version 4.2.6.1. The germline variants were scored using GATK CNNScoreVariants with the option ‘--tensor-type read_tensor’ and filtered using FilterVariantTranches with the options ‘--info-key CNN_2D --invalidate-previous-filters true’. The following variants were excluded to obtain potentially significant somatic variants: 1) variants that did not pass the filter, 2) germline variants, 3) variants with a depth of less than 40, and 4) variants with an allele fraction of less than 0.20 in all tumor samples. The potentially significant somatic variants estimated to likely alter protein behaviour (i.e. variants with HIGH or MODERATE impact from SnpEff) were used as a final set of variants.

Structural variants (SVs) were called for each tumor sample using Manta version 1.6.0 in the tumor-normal mode ^4^. The SVs were annotated using SnpEff version 5.1d ^3^. The following criteria were applied to retain potentially significant SVs: 1) the SV passed the Manta filter and 2) the number of split reads supporting the SV was > 1. The SVs with breakpoint positions matching those of the significant SVs were rescued from the excluded SVs. The potentially significant SVs with HIGH or MODERATE impact from SnpEff were used as a final set of SVs.

Copy number variations (CNVs) were detected using Control-FREEC v11.6 ^5^ with the matched normal sample. The configuration file included the following parameters: ploidy = 2, contaminationAdjustment = TRUE, minimalSubclonePresence = 15, gemMappabilityFile = out100m2_hg38.gem, sex=XY and window = 50000. The dbSNP (build 155) VCF file was supplied for both makePileup and SNPfile options.

**Intra-tumor heterogeneity analysis**

Intra-tumor heterogeneity was inferred using Canopy v1.3.0 ^6^. Somatic single nucleotide alterations (SNAs) were identified using GATK Mutect2, as described in the Supplemental Methods, and used as the input SNA set for Canopy. Heterozygous germline variants in tumor samples were detected using GATK Mutect2 and HaplotypeCaller. Copy number alterations (CNAs) were estimated in each tumor sample using FALCON v0.2 ^7^. Common CNA intervals across tumor samples were identified, and major and minor copy numbers for each sample were assigned to the overlapping common intervals. CNA intervals were used as the input CNA set for Canopy if they met the following criteria: 1) length ≥ 5.5 million bp, 2) maximum difference in major copy number across samples ≥ 0.3, and 3) maximum difference in minor copy number across samples ≥ 0.3. MCMC sampling was performed using canopy.sample with the options “K=3:6, numchain=20, max.simrun=50000, min.simrun=10000, writeskip=200, C=NULL, epsilonM=0.01, epsilonm=0.01, cell.line=FALSE”. The optimal number of subclones (optK) was determined using canopy.BIC with the options “burnin=50, thin=5”. The phylogenetic trees were obtained using canopy.post and canopy.output.

### **Single-cell RNA-sequencing (scRNA-seq) analysis**

Cryopreserved cell samples were carefully thawed and immediately applied for single-cell RNA-seq analysis; for which the target capture of 10,000 cells with more than 90% viability were used. Single cells were subjected to the Chromium Instrument (10x Genomics) to generate single-cell gel beads in emulsion, followed by cDNA synthesis, amplification and library construction using the Chromium Single Cell 3′ Reagent Kit v3 (10x Genomics, CA, USA) according to the manufacturer’s instructions. The final library was amplified and sequenced using the P5 and P7 primers in a DNBseq at BGI Genomics. Cell Ranger v6.0.0 (10X Genomics) was used to align reads to the pre-built GRCh38 reference genome. The molecule information files were combined using cell ranger aggr with --normalize=none --nosecondary options. The R package Seurat v4.0.0 was used for downstream analysis. Cells with UMI counts less than 1500, fewer than 700 genes detected, or ≥ 25% of reads mapped to mitochondrial genes were removed. Read counts were normalized using the Seurat SCTransform function. Variation due to mitochondrial RNA ratio and cell cycle phase scores were regressed out. The data were integrated based on the 3,000 most highly variable genes using the Seurat integration procedure (reciprocal PCA-based integration). Principal component analysis was performed using the Seurat RunPCA function. The top 50 principal components were used for uniform manifold approximation and projection (UMAP) and cell clustering. Cell clustering was performed with a resolution of 0.8. Differentially expressed genes were estimated using the Seurat FindAllMarkers function. Tumor cell clusters were identified based on CD138 (SDC1) expression.**Supplemental Figures**


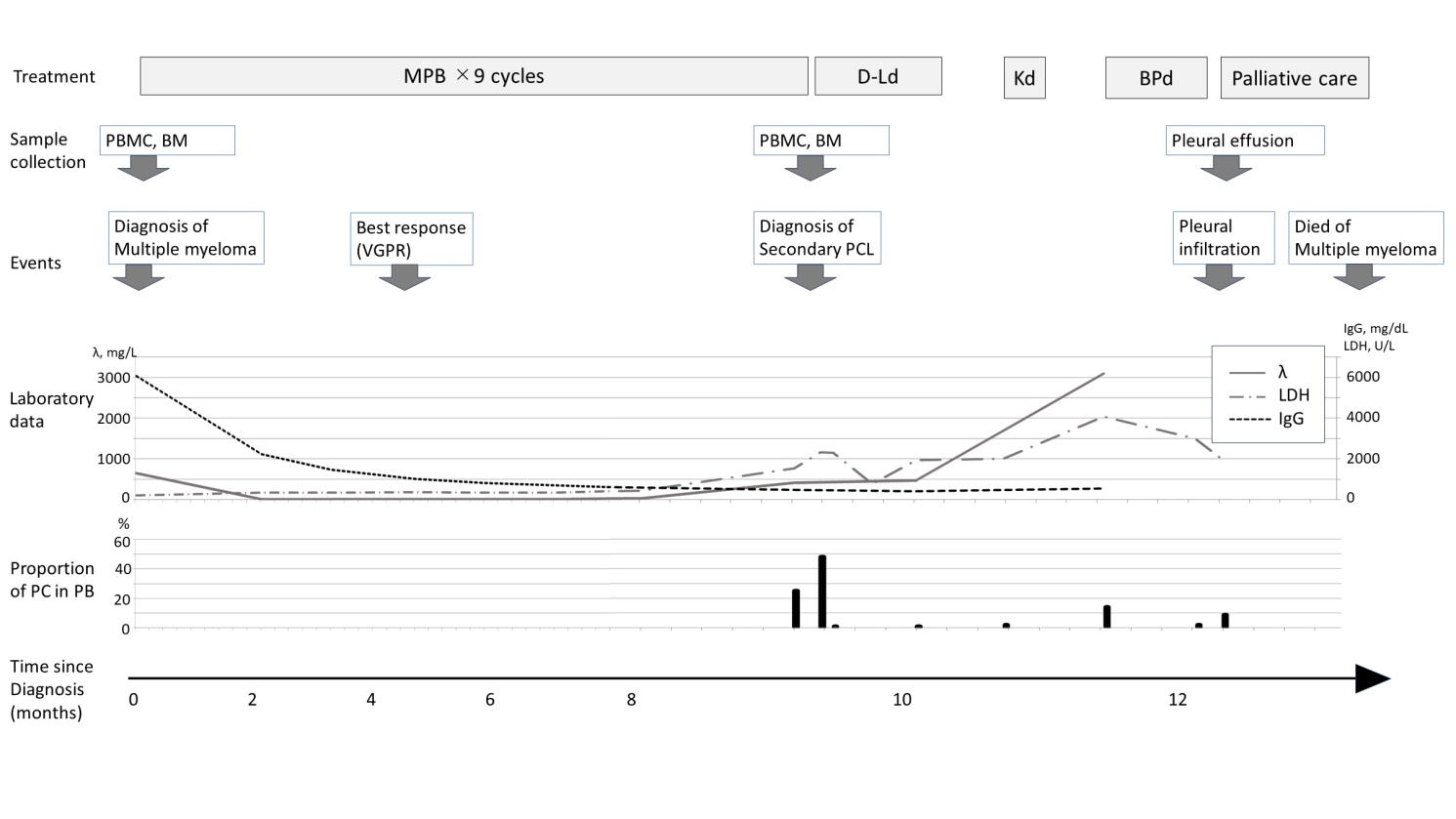


**Figure S1.** Clinical course of the patient

Abbreviations: MPB, melphalan, prednisolone, and bortezomib; D-Ld, daratumumab, lenalidomide and dexamethasone; Kd, carfilzomib and dexamethasone; BPd, bortezomib, pomalidomide and dexamethasone; PBMC, peripheral blood mononuclear cell; BM, bone marrow; VGPR, very good partial response; PC, plasma cell; PCL, plasma cell leukemia; PB, peripheral blood; LDH, lactate dehydrogenase


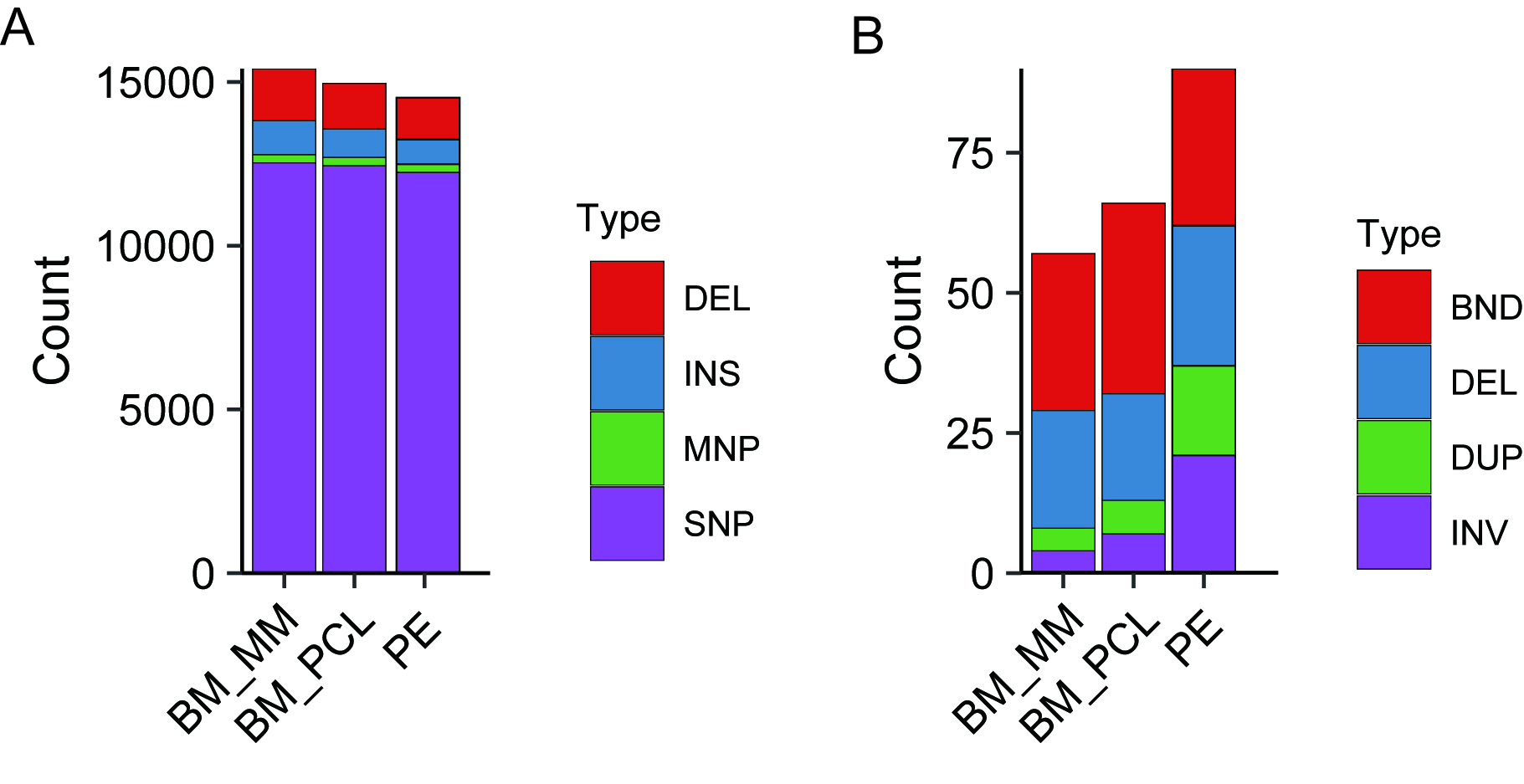


**Figure S2.** Summary of whole-genome sequencing results¶
A: Total number of genetic mutations detected in each sample

B**:** Total number of structural variants estimated in each sample

¶Data for the PB_PCL sample are not shown, as contamination with normal mononuclear cells led to an artificially reduced count of structural variants and genetic mutations.

Abbreviations**:** BM, bone marrow; PCL, plasma cell leukemia; PB, peripheral blood; PE, pleural effusion; bnd, breakend; del, deletion; dup, duplication; inv, inversion; ins, insertion; mnp, multi-nucleotide polymorphism; snp, single-nucleotide polymorphism


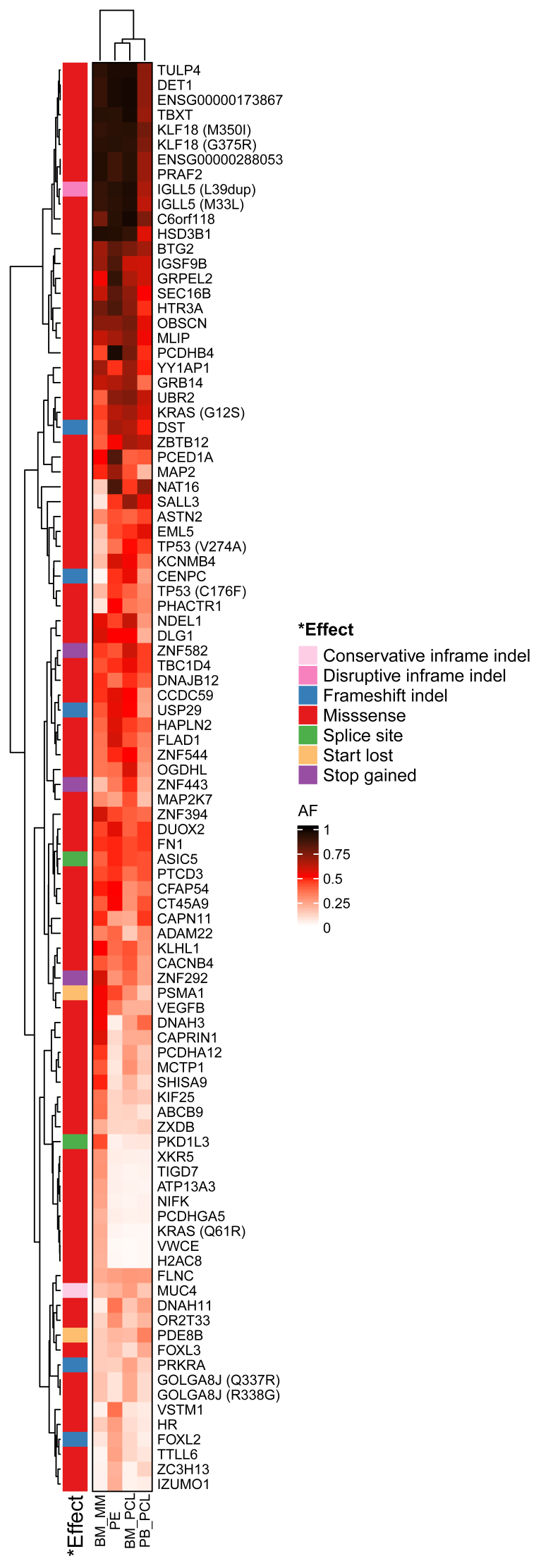


**Figure S3.** Mutational profile of each sample, obtained by whole genome sequencing

Germline variants identified in the peripheral blood sample at the time of initial multiple myeloma diagnosis were evaluated using whole-genome sequencing. These germline variants were excluded from the mutation analysis of each tumor sample.

Abbreviations: PE; pleural effusion, BM_PCL; bone marrow sample obtained at the development of PCL, PB_PCL; peripheral blood sample obtained at the development of PCL, BM_MM; bone marrow sample obtained at the initial diagnosis of multiple myeloma, AF; allele frequency


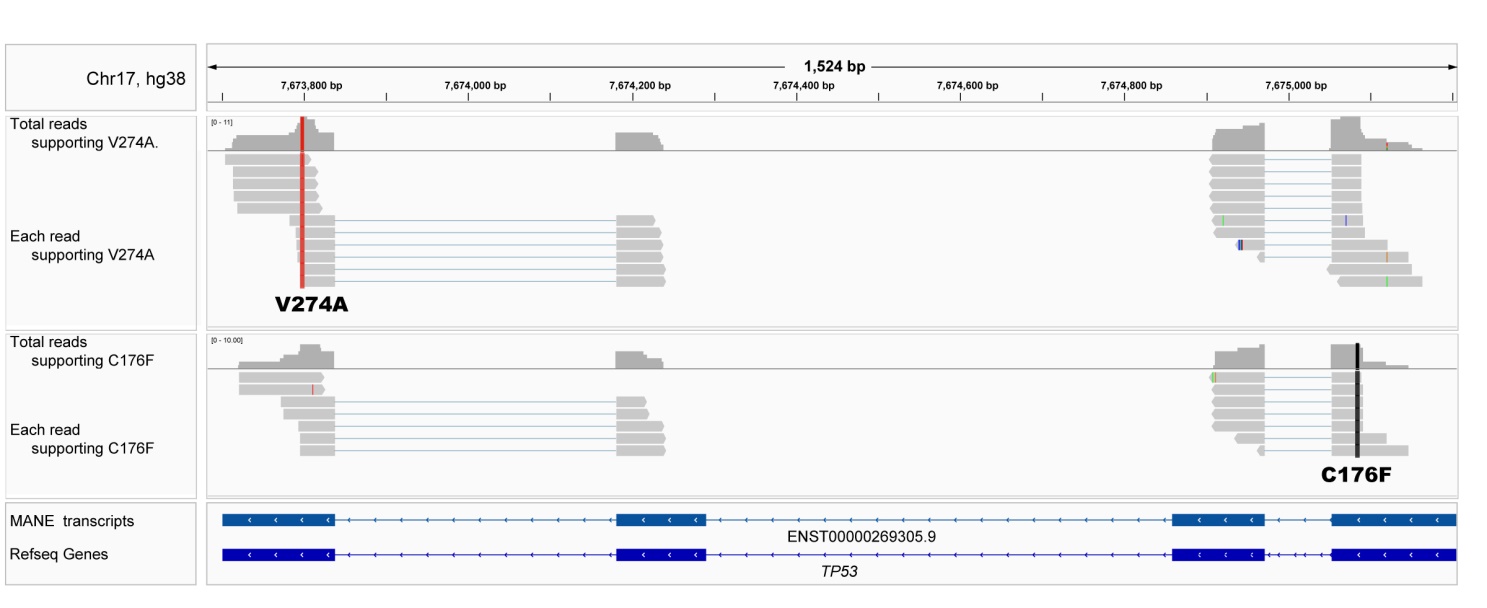


**Figure S4.** RNA sequencing analysis showing the coverage and aligned reads at *TP53* locus in the pleural effusion sample

Each of these reads, which were derived from the same transcript transcribed from one allele, had only one mutation (either V274A or C175F).

## **Supplemental Reference**

[1] Chiang C, Layer RM, Faust GG, et al. SpeedSeq: ultra-fast personal genome analysis and interpretation. Nat Methods. 2015; 12: 966-8.

[2] van der Auwera G, O'Connor BD. Genomics in the Cloud: Using Docker, GATK, and WDL in Terra: O'Reilly Media, Incorporated, 2020

[3] Cingolani P, Platts A, Wang le L, et al. A program for annotating and predicting the effects of single nucleotide polymorphisms, SnpEff: SNPs in the genome of Drosophila melanogaster strain w1118; iso-2; iso-3. Fly (Austin). 2012; 6: 80-92.

[4] Chen X, Schulz-Trieglaff O, Shaw R, et al. Manta: rapid detection of structural variants and indels for germline and cancer sequencing applications. Bioinformatics. 2016; 32: 1220-2.

[5] Boeva V, Popova T, Bleakley K, et al. Control-FREEC: a tool for assessing copy number and allelic content using next-generation sequencing data. Bioinformatics. 2012; 28: 423-5.

[6] Jiang Y, Qiu Y, Minn AJ, Zhang NR. Assessing intratumor heterogeneity and tracking longitudinal and spatial clonal evolutionary history by next-generation sequencing. Proc Natl Acad Sci U S A. 2016; 113: E5528-37.

[7] Chen H, Bell JM, Zavala NA, Ji HP, Zhang NR. Allele-specific copy number profiling by next-generation DNA sequencing. Nucleic Acids Res. 2015; 43: e23.
